# Self-Transcendent Aspirations and Life Satisfaction: The Moderated Mediation Role of Gratitude Considering Conditional Effects of Affective and Cognitive Empathy

**DOI:** 10.3389/fpsyg.2020.02105

**Published:** 2020-08-25

**Authors:** Xavier Oriol, Jesús Unanue, Rafael Miranda, Alberto Amutio, César Bazán

**Affiliations:** ^1^Faculty of Education and Social Science, Universidad Andrés Bello, Santiago, Chile; ^2^Programa de Doctorado en Educación y Sociedad, Facultad de Educación y Ciencias Sociales, Universidad Andrés Bello, Santiago, Chile; ^3^Department of Psychology, Universidad Continental, Lima, Peru; ^4^Faculty of Educación, Universidad Peruana de Ciencias Aplicadas, Lima, Peru

**Keywords:** self-transcendent aspirations, gratitude, cognitive empathy, affective empathy, life satisfaction

## Abstract

Life aspirations are considered one of the most relevant components for human beings to give meaning and purpose to their existence. Different studies emphasized the relevance of intrinsic life aspirations to promote life satisfaction. However, few studies analyze the specific role of the intrinsic aspirations that have been recently categorized as self-transcendent. Self-transcendent aspirations are focused on helping others and improving society and, consequently, are considered aspirations whose purpose transcends oneself. In this sense, the objective of this study is to observe how self-transcendent aspirations are related to life satisfaction through dispositional gratitude. Additionally, we aim to study the moderating role of cognitive and affective empathy. There were 1,356 students (mean age = 21.5, standard deviation = 2.35 years) who took part in a scholarship program funded by the Education Ministry of Peru (PRONABEC), of which 57.7% were men and 42.3% were women. Results show a strong relationship between self-transcendent aspirations, gratitude, and cognitive and affective empathy. In the mediation analysis, an indirect effect of self-transcendent aspirations is observed on life satisfaction via gratitude. However, the moderated mediation showed that the addition of cognitive and affective empathy conditions the mediation effect. In this way, cognitive empathy has a significant interaction in the relationships between self-transcendent aspirations and gratitude, and between self-transcendent aspirations and life satisfaction. Results are discussed to emphasize the relevance of the mediating and moderating mechanisms considered in this study for the understanding of how self-transcendent life aspirations may promote life satisfaction.

## Introduction

Life aspirations reflect long-term goals and are considered one of the most relevant components for human beings to give meaning and purpose to their existence (Steger et al., 2006). Aspirations enable prospective thinking (about the future) and allow us to direct our actions and organize our cognitive and affective processes in the present time (Seligman et al., 2013; Baumeister et al., 2016).

According to the self-determination theory (SDT) proposed by Ryan and Deci (2000), life aspirations drive us and lead our choices and lifestyles. To measure these aspirations, Kasser and Ryan (1996) developed the Aspiration Index, a scale to assess intrinsic aspirations (personal growth, close relationships, community involvement, and physical health), as well as extrinsic aspirations (popularity, financial success, and image). Different studies show that intrinsic aspirations are associated with the “own nature” of the individual and therefore entail high probabilities of increasing well-being indicators such as positive affect and vitality (Lekes et al., 2010; Hope et al., 2016), whereas extrinsic aspirations are related to materialistic motives and have been linked to feelings of frustration and decreased life satisfaction (Dittmar et al., 2014; Unanue et al., 2014).

Grouzet et al. (2005) created a new classification of these life aspirations, which, besides the categories intrinsic and extrinsic, makes a distinction between self-transcendent and physical goals. According to these authors, self-transcendent goals are intrinsic aspirations that are considered prosocial, as they imply connecting with others and going beyond selfish concerns. More recently, Martela et al. (2019) conducted a study with two samples of adults to verify, using a circular stochastic modeling approach, if, in addition to the intrinsic and extrinsic categories, aspirations could also be categorized within the physical self vs. transcendence axes. The results confirmed that the relationship between aspirations can be described as a set of variables distributed along the circumference of a circle.

### Self-Transcendent Aspirations and Subjective Well-Being

According to SDT, understanding the role played by life aspirations in subjective well-being (SWB) is fundamental (Kasser and Ryan, 1993; Bandura, 1997; Martela et al., 2019). Different studies have related intrinsic aspirations to different indicators of well-being (for review Ryan and Deci, 2017). However, few studies have linked these aspirations to the affective and cognitive components of SWB, as developed by Diener (1984). As such, SWB comprises a cognitive component, which is measured by asking people how satisfied they are with their lives (life satisfaction), and an affective component, which refers to the presence of positive affect or to the absence of negative affect, and it is assessed by inquiring about the experience of specific emotions during the previous day or week (Jebb et al., 2020).

Some of the research on the relationship between intrinsic aspirations and the affective and cognitive components of SWB shows a positive and significant relationship with positive affect (Kasser and Ryan, 1996; Romero et al., 2012; Martela et al., 2019). Also, a positive and significant relationship was found between intrinsic aspirations and life satisfaction in the studies by Romero et al. (2012) and Nishimura and Suzuki (2016); however, a negative and significant relationship was discovered in the work conducted by Allan and Duffy (2014). More recently, Martela et al. (2019) observed, by means of a regression analysis, that intrinsic aspirations have a positive and significant relationship with life satisfaction, but the significant effect disappeared when considering the participant’s mean scores for life aspiration (calculated through the mean of the importance rated to the 13 intrinsic and extrinsic aspirations for each of the participants).

If we consider aspirations categorized by their axes, self-transcendence vs. physical self (Grouzet et al., 2005; Martela et al., 2019), and their relationship with the components of well-being, specifically, the intrinsic aspiration of community contribution (i.e., generativity and helping others), which is the closest to the self-transcendence axis (Martela et al., 2019), has been linked to both positive affect and life satisfaction, as well as to the global component of SWB (Martela and Ryan, 2016; Martela et al., 2019). Concretely, the items of this type of self-transcendent aspiration refer to the importance of objectives related to helping others and improving society and consequently are considered aspirations whose purpose transcends oneself.

In a recent review article published by Diener et al. (2018), the authors emphasize the importance of considering fundamental aspects like aspirations related to social connection with others as predictors in SWB measures. Much of the existing literature indicates that people’s motivation to build and maintain social relationships is essential for survival and well-being (Leary and Baumeister, 2017). Additionally, when human beings provide support to others, this may give them a sense of meaning that is associated with positive affect and life satisfaction (Post, 2005; Siedlecki et al., 2014).

In conclusion, many of the previous studies based on SDT (Ryan and Deci, 2000) that relate to life aspirations have found that intrinsic aspirations are positively related to different well-being indicators. Furthermore, recently, interest in the study of intrinsic aspirations that promote self-transcendence and their relationship with the different components of SWB has grown (Grouzet et al., 2005; Martela et al., 2019). Specifically, this research work seeks to demonstrate the relationship between self-transcending life aspirations that represent long-term goals focused on helping the community and that transcend the self, and the cognitive component of well-being (i.e., life satisfaction), while considering different mechanisms that mediate and moderate this relationship. Setting long-term goals is essential for human beings, but progressing toward them is what contributes to increasing satisfaction with life (Diener, 1998). Therefore, we believe that a better understanding on this subject is necessary, because different mechanisms, such as gratitude and empathy, are fundamental to maintain long-lasting interpersonal connections and promote prosocial behavior, while at the same time they intervene in the relationship between self-transcendent aspirations and satisfaction with life.

### Gratitude as a Potential Mediator

Over the past years, there has been increasing interest in the role of transcendence emotions such as gratitude in life satisfaction (Wood et al., 2008; Robustelli and Whisman, 2018). Gratitude falls within the categories of self-transcendent emotions (Van Cappellen et al., 2016; Stellar et al., 2017) and “other-praising emotions,” according to the moral categories developed by Haidt (2003). This emotion is experienced when a person is helped by others. It is usually defined as a state or as a trait (Emmons and McCullough, 2004) and can act as a driver to enhance the welfare of others (Bartlett and DeSteno, 2006; Campos et al., 2013).

Self-transcendent emotions such as gratitude are considered emotions that promote the development and maintenance of long-term social relationships (Stellar et al., 2017; DeSteno, 2018). Different studies have demonstrated that gratitude stimulates prosocial behavior toward others (for a review, see McCullough et al., 2001). Particularly, people who experience gratitude have more possibilities of helping strangers (Bartlett and DeSteno, 2006). In this sense, gratitude has been related to prosocial conducts and social support, which also promote life satisfaction (Algoe, 2012; Alkozei et al., 2018).

Additionally, gratitude is oriented toward noticing positive outcomes in life and promotes positive experiences and, specifically, positive emotions in daily life, which broaden the scope of thought and increase satisfaction with one’s life (Emmons et al., 2019). So, when people feel constantly grateful for the situations happening and these are seen as a gift, they are more likely to increase their personal resources to face daily experiences in a more positive way (Unanue et al., 2019). Along the same line, the broaden-and-build theory developed by Fredrickson (2004) suggests that gratitude can broaden an individual’s awareness of positivity and contribute to the accumulation of resources for achieving life goals, thus increasing life satisfaction.

Like other self-transcendent emotions such as awe and compassion, gratitude requires a personal view that transcends the own self to focus on others (Stellar et al., 2017). The elicitation of this type of emotions, as it occurs with other emotions, depends on the relevance that social and cultural contexts attribute to self-focused concerns (Mesquita et al., 2016). In a recent study, De Leersnyder et al. (2018) observed significant differences in the emotional experience of self-focused and other-focused concerns. Consequently, it is important to explore how these long-term life aspirations can promote emotions such as gratitude and how gratitude, in turn, can facilitate other-oriented motivation. So, the first goal of this study was to observe how self-transcendent aspirations are related to life satisfaction through dispositional gratitude. Taking into account the aforementioned, we hypothesize that:

H1. Self-transcendent aspirations will be positively related to life satisfaction through gratitude.

Specifically, we expect that gratitude (i.e., directing cooperation to others and fostering long-term relationships) will mediate the relationship between self-transcendent aspirations and life satisfaction.

### The Moderation Role of Cognitive and Affective Empathy

Empathy is considered a complex affective and cognitive process of understanding and feeling others’ emotions (Baron-Cohen and Wheelwright, 2004; Jolliffe and Farrington, 2006). Empathy is a primary mechanism fundamental for interpersonal connection and therefore acts as a prosocial behavior driver through sensitivity to emotional signals from others (for review, see Eisenberg, 2010; De Waal, 2012).

Different studies have associated the lack of empathy with violence, aggression, and other interpersonal problems (Blair et al., 2005; Mitsopoulou and Giovazolias, 2015), whereas empathy has been linked to positive interpersonal relationships and friendship (Durlak et al., 2011; Hayes and Ciarrochi, 2015). Nevertheless, other studies have hardly found a weak relationship between empathy and aggressiveness (for a review, see Vachon et al., 2014).

An important aspect of empathy seems to be that the focus of concern has to be others in order to prevent excessive personal anguish (Eisenberg et al., 1996). Previous studies have shown that being empathetic with others is related to a tendency to prosocial behavior, social closeness, and life satisfaction (Morelli et al., 2015). However, occasionally, in the absence of the cognitive mechanisms conducive to perspective-taking, an excess of affective empathy can cause personal distress and suffering (Eisenberg et al., 2006; Blanco-Donoso et al., 2017; Amutio et al., 2018) and lead to a decrease in prosociality (Decety and Lamm, 2009; Decety and Yoder, 2016). This fact has sparked a debate on how to separate the mechanisms of affective and cognitive empathy (Cuff et al., 2016).

Both mechanisms (affective and cognitive) have been speculated to be relevant to life satisfaction. However, difficulties for perspective-taking when facing affective stimuli can cause increased negative affect, including distress and clinical symptomatology (Morelli et al., 2015; Lachmann et al., 2018). More generally, people who experience difficulties to assess and cope with a stressful situation feel anguished, and this impairs their prosocial qualities, including empathy (Batson, 1991). Following this perspective, different studies have pointed to the need of understanding that empathy is a general term to describe several forms in which people respond to one another, including sharing and thinking about the feeling of others (for a review, see Zaki, 2020). This implies that empathy can encourage ways of worrying about and experiencing the same emotions as others, but without mechanisms that target cognitively and rationally perspective-taking, suffering and emotional wear may occur (Bloom, 2017a, b). In this sense, the definition of cognitive empathy (perspective-taking) implies a more rational processing that promotes sensitivity to justice for others, as well as the endorsement of moral rules, which is also the case with emotions such as compassion (Decety and Yoder, 2016; Bloom, 2017b).

Aspirations that imply life goals can generate frustration in people if they are not encompassed by achievements or materialization (Ryan and Deci, 2000). Therefore, in the case of self-transcendent aspirations, as already noted, the experience of dispositional gratitude is considered a fundamental mediating mechanism for relating these aspirations to life satisfaction, thus preventing them to cause frustration. Nevertheless, promoting gratitude implies building reciprocal constant relationships in terms of self-benefit and benefactor-cost (Emmons and McCullough, 2004; Van Cappellen et al., 2016). In addition, human beings have neural mechanisms, such as empathy and mentalization, which enable the simulation of others’ mental states, which gives rise to gratitude and reciprocity (Yu et al., 2018). In this sense, the literature on this subject leads us to think that high and low scores for both forms of empathy can condition the relationship between aspirations and the constant experience of gratitude. Without high empathy, attributing generous intentions to others is difficult, and this, in turn, affects the experience of gratitude (Bloom, 2017b; Stern et al., 2019). Furthermore, as suggested by the literature, the experience of affective empathy can take a high toll in people (Zaki, 2020), generating more distress and suffering and consequently impairing the experience of gratitude.

**FIGURE 1:**
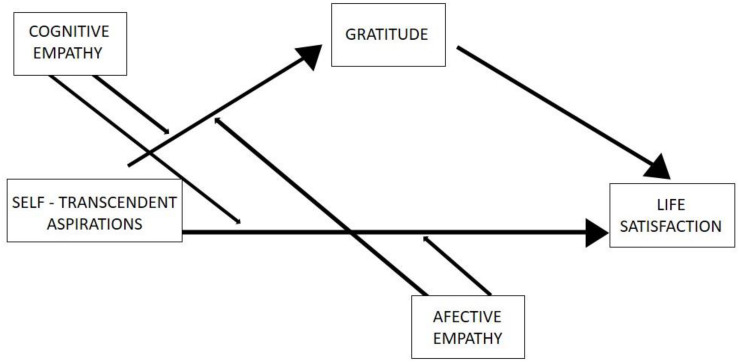
Conceptual model: moderated mediation between self-transcendent aspirations and life satisfaction.

In the same line, we also want to demonstrate how both forms of empathy moderate the relationship between self-aspirations and life satisfaction. From an evolutional perspective, empathy promotes the connection between people (Bloom, 2017a), which we believe is fundamental to achieve high levels of SWB in the search of goals that transcend self-related concerns to common good. Without a high ability to understand other people’s thoughts and feelings, it would be hard for people to experience a feeling of well-being in their connection with others or prosocial and altruistic behaviors toward others (Grühn et al., 2008). Additionally, as already mentioned, the differences in the experience of affective and cognitive empathy in the well-being perception (Bloom, 2017b; Zaki, 2020) lead us to think that both forms of empathy can condition the relationship between aspirations and life satisfaction differently. In this sense, it is expected that both forms of empathy will show a moderating effect in the relationship between aspirations and gratitude, and in the relationship between aspirations and life satisfaction. Therefore, the following hypotheses are also formulated (Figure 1):

H2. Cognitive and affective empathy are expected to have a moderating effect on the relationship between self-transcendent aspirations and gratitude. Specifically, all interactions will be significant.

H3. Both forms of empathy are expected to condition the relationship between self-transcendent aspirations and life satisfaction through gratitude. Therefore, significant moderated mediation indexes are expected for the conditioned effects, considering both forms of empathy.

## Materials and Methods

### Participants

The sample is composed of 1,356 university students [mean age = 21.5, standard deviation (*SD*) = 2.35 years], of which 57.7% are men and 42.3% women. All of them are part of a total of 40,000 students who belong to a scholarship program financed by the Education Ministry of Peru (PRONABEC). The Ministry of Education conducted this study to verify the indicators related to well-being in university students from this program. In order to obtain an adequate psychometric validation of the global questionnaire (78 items), 1,400 students were randomly selected to participate in the study, of which 1,356 were successfully completed.

A sensitivity power analysis was conducted using G^∗^Power 3.1 (Faul et al., 2009) considering α = 0.05, a desired power of 0.80, and 11 parameters in a moderate mediation model with two linear regressions, each of which had three principal predictors and two interactions. An effect size of *f*^2^ = 0.006 was obtained. Thus, the model with 80% power can detect a predictor with a population effect size of *f*^2^ = 0.006, which is considered a small size by Cohen (1992). The study had the support of an external group of university researchers that helped create the questionnaire and provided insights into the ethical aspects below.

### Procedure

The research team and the Ministry of Education developed the questionnaire, paying special attention to compliance with the ethical standards required for this type of study. To this end, informed consents that explained the objectives, confidentiality, and the voluntary character of the study were created to be signed by participants prior to survey application. Different specialists from the same unit established the protocols for the implementation of the survey. The Research Ethics Committee of the Continental University (Peru) reviewed and approved the ethical suitability of the study considering the ethical recommendations of the Declaration of Helsinki for studies on human beings.

PRONABEC has a virtual platform in INTRANET (private network system), which is a communication system that allows for sending and collecting information from its students. Through this system, the PRONABEC team programmed the online application of the survey. The questionnaire was sent to a randomized sample of students from the program. When the questionnaire was launched on the platform, all selected students received a notification via email so that they could access and complete the survey.

Before filling in the questionnaire, students had to read the research purpose and provide an electronic signature. Students took an average of 30 to 40 min to complete the questionnaire. It is worth mentioning that this survey was available for 2 weeks on the platform with the aim of collecting as much information as possible.

### Measures

#### Self-Transcendent Aspirations

The subscale “community involvement” from the *Aspirations Index* developed by Kasser and Ryan (1996) was used. This subscale asks respondents how important some aspirations related to prosociality are to them (e.g., “To help others improve their lives;” “To help people in need”) in a 1- to 7-point scale (“very important”). For this study, the scale presents a Cronbach α of 0.91.

#### Gratitude

The subscale of gratitude from the Positive Emotion Questionnaire developed by Oros (2014) was used. This questionnaire assesses different dispositional emotions such as gratitude, sympathy, serenity, and satisfaction. The gratitude subscale contains four items related to gratitude experiences in daily life. To answer the questionnaire, students need to answer to which extent they agree or disagree with the statements in a range from 1 (totally disagree) to 5 (totally agree) (e.g., “I am thankful for the things I have;” “I like to be thankful with other people”). Cronbach α for this scale was 0.91.

#### Cognitive and Affective Empathy

The 9-item scale proposed by Soto and Muchotrigo (2015) was used to assess empathy. This measure evaluates the construct for the affective (e.g., “I get sad when I see people crying”) and cognitive dimensions (e.g., “When someone is depressed I use to understand him/her) in a range between 1 (totally disagree) and 5 (totally agree). Cronbach α was 0.86

#### Life Satisfaction

The Brief Multidimensional Students’ Life Satisfaction Scale developed by Seligson et al. (2003) was used. This scale contains five items that deal with different domains related to life satisfaction. The items measure to what extent the students feel “dissatisfied” or “satisfied” with the following aspects of their life: (1) family life, (2) friendships, (3) life at school/institute, (4) self-satisfaction, and (5) the place where they live. This instrument uses a scale from 1 “very dissatisfied” to 7 “very satisfied.” Cronbach α was 0.90.

### Data Analysis

Descriptive analyses were conducted using SPSS 23.0. For mediation and moderate mediation analyses, the models 4 and 10 of the macro Process 3.2.01 proposed by Preacher and Hayes (2008) for SPSS were used, respectively. Mediation indirect effects between the hypothesized variables were computed. Results will reflect statistically significant indirect effects by testing the confidence interval (CI) derived from 10,000 bootstrap repetitions at 95% to verify the mediation effect. The indirect effect is significant if the CI does not contain the value zero (Hayes, 2017). Similarly, a moderate mediation analysis between the hypothesis variables was carried out, evaluating the indexes of partial moderated mediation. These indices “quantify the rate of change in the indirect effect of independent variables as one moderator changes but the other is held constant” (Hayes, 2018, p. 11), which will show a statistically significant indirect effect if the CI does not contain the value zero (Hayes, 2018). It must be noted that the estimation of the parameters for each model was carried out by means of ordinary least squares regression. Finally, we ruled out the gender and age control variables because they are not significantly correlated with life satisfaction.

## Results

### Descriptive Statistics

First, means, *SD*s, and bivariate correlations between all the variables are presented using SPSS 23.0 (Table 1). High, positive, and significant correlations can be observed across all the variables above, with gratitude and cognitive empathy standing out (*r* = 0.56, *p* = 0.000).

**TABLE 1:** Means, standardized deviations, and correlations between all variables.

		Mean	*SD*	1	2	3	4	5	6	7
1.	Gender	1.42	0.49							
2.	Age	21.53	2.35	−0.21**						
3.	Gratitude	3.37	0.62	0.07**	0.02					
4.	ST aspirations	5.16	0.91	0.13**	0.01	0.52**				
5.	Affect empathy	2.44	0.90	0.06*	0.04	0.26**	0.16**			
6.	Cognitive empathy	2.90	0.66	0.04	0.01	0.56**	0.40**	0.53**		
7.	Life satisfaction	4.69	1.42	−0.01	0.01	0.32**	0.19**	0.08**	0.20**	

*p < 0.05, **p < 0.01.

### Mediation Analysis

**FIGURE 2:**
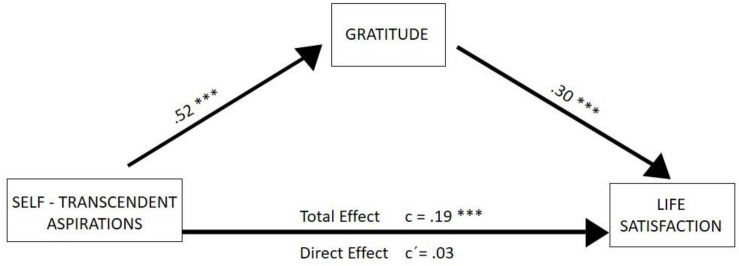
Mediation model for self-transcendent aspirations and life satisfaction through gratitude. Standardized regression coefficients are presented. *p < 0.05, **p < 0.01, ***p < 0.001.

The results of the first model (Figure 2), with dispositional gratitude as a possible mediating variable, self-transcendent aspirations as an independent or predictor variable, and life satisfaction as a dependent variable, show that self-transcendent aspirations have an direct effect that is not statistically significant on life satisfaction (β = 0.03, *p* > 0.05). However, when introducing gratitude, we observe that it has a statistically significant indirect effect (β = 0.16, 95% CI = 0.107–0.205). Thus, the relationship between self-transcendent aspirations and life satisfaction is interpreted as being mediated by dispositional gratitude. Values of *R*^2^ fluctuated from 0.27 to 0.36 (*p* = 0.000).

### Moderate Mediation Analysis

For the second model (Figure 1), taking self-transcendent aspirations as the independent variable, keeping life satisfaction as the dependent variable, and gratitude as a mediator, cognitive empathy and affective empathy are introduced as possible moderators of this mediation.

For the model that takes gratitude as dependent variable (Table 2), the construct was positively and significantly predicted by self-transcendent aspirations (β = 0.47, *p* < 0.001) and cognitive empathy (β = 0.77, *p* < 0.001), whereas it was negatively and significantly affected by the interaction between self-transcendent aspirations and cognitive empathy (β = −0.08, *p* < 0.01). However, affective empathy predicts gratitude positively but not significantly (β = 0.08, *p* > 0.05), and the same is true for the interaction with self-transcendent aspirations (β = −0.02, *p* > 0.05). Direct conditional effects are shown in Table 2 for the case where both moderators assume values of −1 *SD*, mean, and +1 *SD* and their combinations. The effects are significant at all levels.

**TABLE 2:** Conditional direct effects between self-transcendent aspirations and gratitude moderated by affect and cognitive empathy.

Predictor	β	SE	Lower 95% BootLLCI	Upper 95% BootULCI
**DV = gratitude as dependent variable model**				
Self-transcendent aspirations	0.47*	0.04	0.40	0.53
Cognitive empathy	0.77*	0.1300	0.52	1.02
Self-transcendent aspirations × cognitive empathy	−0.08**	0.02	−0.12	−0.03
Affect empathy	0.08	0.13	−0.17	−0.33
Self-transcendent aspirations × affect empathy	−0.02	0.02	−0.07	0.03

**Conditional direct effect at different values of the moderator**				

**Moderator values**	**β**	**BootSE**	**BootLLCI**	**BootULCI**

−1 *SD* cognitive empathy and −1 *SD* affect empathy	0.27*	0.02	0.23	0.30
−1 *SD* cognitive empathy and mean affect empathy	0.25*	0.02	0.21	0.29
−1 *SD* cognitive empathy and +1 *SD* affect empathy	0.23*	0.04	0.16	0.30
Mean cognitive empathy and −1 *SD* affect empathy	0.22*	0.03	0.16	0.27
Mean cognitive empathy and mean affect empathy	0.20*	0.02	0.17	0.23
Mean cognitive empathy and +1 *SD* affect empathy	0.18*	0.03	0.13	0.23
+1 *SD* cognitive empathy and −1 *SD* affect empathy	0.17*	0.04	0.09	0.25
+1 *SD* cognitive empathy and mean affect empathy	0.15*	0.03	1.0	0.20
+1 *SD* cognitive empathy and+1 *SD* affect empathy	0.13*	0.02	0.09	0.17

*p < 0.001, **p < 0.01.

**FIGURE 3:**
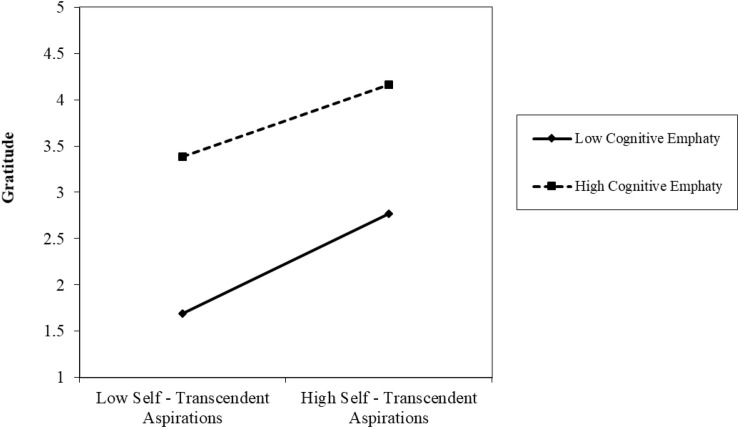
Interaction between self-transcendent aspirations and cognitive empathy over gratitude.

Figure 3 shows the interaction between self-transcendent aspirations and cognitive empathy over gratitude. Students with low self-transcendent aspirations and low cognitive empathy present significant differences in gratitude compared to those with low self-transcendent aspirations and high cognitive empathy. However, the difference is smaller between students with high self-transcendent aspirations and low cognitive empathy, and students with high self-transcendent aspirations and high cognitive empathy. The slope in the case of low cognitive empathy is also slightly steeper than the slope of high cognitive empathy, but a higher level of dispositional gratitude is observed in both cases for high levels of the self-transcendent aspirations.

The results of the model with life satisfaction as a dependent variable show that affective empathy does not significantly influence life satisfaction (β = 0.06, *p* > 0.05), but again self-transcendent aspirations are observed to have a positive and statistically significant effect (β = 0.63, *p* < 0.005), as well as gratitude (β = 0.56, *p* < 0.001) and cognitive empathy (β = 1.18, *p* < 0.005). With respect to the interactions in this model, only cognitive empathy and self-transcendent aspirations present a negative and significant effect on life satisfaction (β = −0.21, *p* < 0.005). Direct conditional effects are shown in Table 3. For the case in which both moderators assume values of −1 *SD*, mean, and +1 *SD* and their combinations, only three out of nine effects are significant: −1 SD cognitive empathy and −1 *SD* affect empathy (β = 0.12, 95% CI = 0.11–0.23), +1 *SD* cognitive empathy and mean affect empathy (β = −0.18, 95% CI = −0.32–0.03), and +1 *SD* cognitive empathy and +1 *SD* affect empathy (β = −0.20, 95% CI = −0.31 to −0.07).

**TABLE 3:** Conditional direct effects between self-transcendent aspirations and life satisfaction moderated by affect and cognitive empathy.

Predictor	β	SE	Lower 95% BootLLCI	Upper 95% BootULCI
**DV = LS as dependent variable model**				
Self-transcendent aspirations	0.63*	0.11	0.42	0.84
Gratitude	0.56*	0.08	0.41	0.71
Cognitive empathy	1.18**	0.37	0.46	1.90
Self-transcendent aspirations × cognitive empathy	−0.21**	0.07	−0.34	−0.01
Affective empathy	0.06	0.36	−0.65	0.76
Self-transcendent aspirations × affective empathy	−0.02	0.07	−0.15	0.11

**Conditional direct effect at different values of the moderator**				

**Moderator values**	**β**	**BootSE**	**BootLLCI**	**BootULCI**

−1 *SD* cognitive empathy and −1 *SD* affective empathy	0.12***	0.06	0.01	0.23
−1 *SD* cognitive empathy and mean affect empathy	0.10	0.06	−0.01	0.21
−1 SD cognitive empathy and +1 *SD* affect empathy	0.08	0.10	−0.12	0.28
Mean cognitive empathy and −1 *SD* affect empathy	−0.02	0.08	−0.18	0.15
Mean cognitive empathy and mean affect empathy	−0.04	0.05	−0.13	0.06
Mean cognitive empathy and +1 *SD* affect empathy	−0.06	0.07	−0.20	0.08
+1 *SD* cognitive empathy and −1 *SD* affect empathy	−0.16	0.12	−0.39	0.08
+1 *SD* cognitive empathy and mean affect empathy	−0.18***	0.08	−0.32	−0.03
+1 *SD* cognitive empathy and +1 *SD* affect empathy	−0.20**	0.06	−0.32	−0.07

*p < 0.001, **p < 0.01, ***p < 0.05.

**FIGURE 4:**
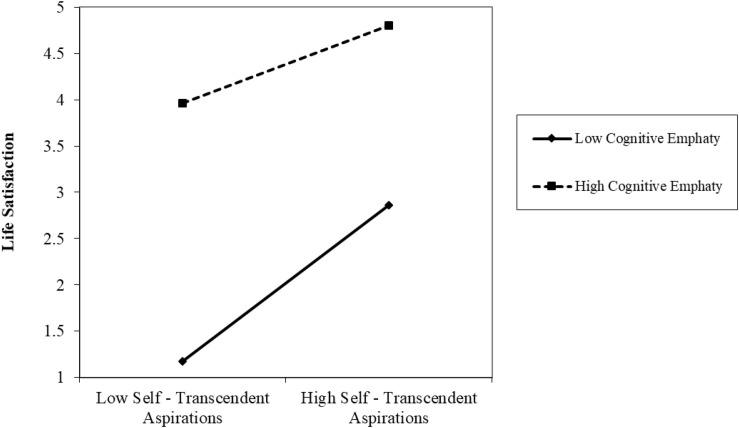
Interaction between self-transcendent aspirations and cognitive empathy over life satisfaction.

Figure 4 shows the effect of self-transcendent aspirations and cognitive empathy over life satisfaction, with participants who report higher levels of cognitive empathy and self-transcendent aspirations having increased levels of life satisfaction. Moreover, as self-transcendent aspirations increases, so does life satisfaction, regardless of the level of cognitive empathy. However, the regression slope is steeper for people with lower cognitive empathy.

Conditional indirect effects are presented in Table 4. In all cases, they are statistically significant. The index of partial moderated mediation was only significant for cognitive empathy (β = −0.04, 95% CI = −0.08 to -0.01). Therefore, only cognitive empathy moderates the mediating role of gratitude in the effect of self-transcendent aspirations on life satisfaction. Values of *R*^2^ fluctuated from 0.13 to 0.44 (*p* = 0.000).

**TABLE 4:** Conditional indirect effects between self-transcendent aspirations and life satisfaction through gratitude and moderated effects of affective and cognitive empathy.

Conditional indirect effect at different values of the moderator	β	BootSE	Lower 95% BootLLCI	Upper 95% BootULCI
**Moderator values**				
−1 *SD* cognitive empathy and −1 *SD* affect empathy	0.15*	0.02	0.10	0.19
−1 *SD* cognitive empathy and mean affect empathy	0.14*	0.02	0.10	0.18
−1 *SD* cognitive empathy and +1 *SD* affect empathy	0.13*	0.03	0.08	0.19
Mean cognitive empathy and −1 *SD* affect empathy	0.12*	0.03	0.07	0.17
Mean cognitive empathy and mean affect empathy	0.11*	0.02	0.08	0.15
Mean cognitive empathy and +1 *SD* affect empathy	0.10*	0.02	0.06	0.14
+1 *SD* cognitive empathy and −1 *SD* affect empathy	0.09*	0.03	0.04	0.15
+1 *SD* cognitive empathy and mean affect empathy	0.08*	0.02	0.05	0.12
+1 *SD* cognitive empathy and +1 *SD* affect empathy	0.07*	0.02	0.05	0.11
Index of partial moderated mediation for cognitive empathy	−0.04*	0.02	−0.08	−0.01
Index of partial moderated mediation for affect empathy	−0.01	0.02	−0.04	0.02

*p < 0.05.

## Discussion

Aspirations and expectations for the future imply a type of prospective thinking relevant to the human being, which makes our species different from animals (Baumeister et al., 2016). This study aims to delve into how self-transcendent aspirations can affect the perception of our own life as a whole. Descriptive data obtained in this study show a positive relationship between self-transcendent aspirations and life satisfaction, as well as between aspirations and variables closer to prosociality, such as both forms of empathy and gratitude.

Following our first hypothesis, an indirect effect ofself-transcendent aspirations is observed on life satisfaction through gratitude, thus confirming this hypothesis. Specifically, this mediating effect is total. Additionally, it must be noted that self-transcendent aspirations have a positive and significant direct effect on gratitude. This result underscores the relevance of this type of aspirations, which represent long-term goals that can drive emotions that strengthen and maintain interpersonal relationships in the long term such as gratitude (Stellar et al., 2017). Self-transcendent aspirations as community involvement focus people’s interest not only on themselves, but also on others (Kasser and Ryan, 1996), and in this aspect, our results support the hypothesis that these aspirations are important to guide affective positive experiences induced by gratitude in daily life. Moreover, dispositional gratitude, which is considered a general tendency to respond to others with gratitude or appreciation (McCullough et al., 2002), can be an important mechanism for linking these aspirations to the subjective perception of well-being (i.e., life satisfaction). From a bottom-up perspective, positive and negative daily-life affective experiences contribute to build the cognitive judgment. that people make about their lives as a whole (Kahneman and Riis, 2005). Consequently, the tendency to experience gratitude can promote the accumulation of positive affective experiences that increase life satisfaction. As suggested by Diener (1998) for the well-being of human beings, it is important not only to set long-term goals, but also to achieve them. In this sense, dispositional gratitude, as indicated by our results, may encourage the materialization of self-transcendent aspirations into gratifying actions in daily life that promote the accumulation of positive personal resources, as well as keeping positive interpersonal relationships in the long term, which in turn increases life satisfaction.

Regarding the second hypothesis, high empathy and low cognitive empathy moderate the relationship between self-transcendence aspirations and gratitude. People who score higher in cognitive empathy experience more gratitude than those with lower empathy. Nevertheless, the effect of the interaction is stronger in people with low scores in cognitive empathy than in people with high scores, which implies that this factor has an important effect on the relationship between self-transcendent aspirations and gratitude. These data support the importance of understanding others’ affect for the development of emotions that fall into the category of prosocial emotions and or transcendence emotions (Stellar et al., 2017). In this sense, the development of cognitive empathy seems vital to promote emotions such as gratitude, which are basic to foster prosocial behavior (for review, see Ma et al., 2017). In another vein, affective empathy does not show a moderating effect, which may indicate, as observed in the literature, that cognitive perspective-taking toward others in empathy processes is essential to prevent the anxiety and other types of negative emotions that hinder gratitude (Decety and Lamm, 2009; Decety and Yoder, 2016). So, the second hypothesis is partially confirmed.

In the third hypothesis, we sought to confirm the moderating role of both forms of empathy in the relationship between aspirations and life satisfaction through gratitude. First, a significant interaction of cognitive empathy is observed, but without a moderating effect of affective empathy on the relationship between aspirations and life satisfaction. People with higher cognitive empathy present a positive significant relationship between self-transcendent aspirations and life satisfaction, and a significant effect is observed again on the interaction with people who have lower cognitive empathy. Consequently, the results confirm that, in the relationship with life satisfaction, low empathy also has a negative impact on the association between aspirations and the cognitive component of SWB.

Regarding the moderated mediation, a conditional indirect effect is observed, which takes place when considering both forms of empathy between aspirations and life satisfaction through gratitude. Nevertheless, as commented above, only the moderated mediation index for cognitive empathy is significant. Thus, the third hypothesis is partially confirmed. This underscores the relevance of considering the affective and cognitive mechanisms of empathy separately, as well as the possible role of cognitive empathy in the promotion of life satisfaction and in the prevention of emotional exhaustion.

These results are in agreement with different studies focused on the effects of mindfulness meditation, which indicate that taking perspective of how oneself and others feel in a particular situation is essential to foster interest toward others and, at the same time, protect our own SWB (e.g., Dahl et al., 2015; Van Doesum et al., 2018). Other studies also indicate that affective empathy can increase the levels of stress and anxiety in the long term if it does not lead to a more cognitive perspective (Bloom, 2017b; Blanco-Donoso et al., 2017; Amutio et al., 2018).

As main limitations of this study, data presented are cross-sectional, and thus, it would be relevant to obtain longitudinal data to better define the causality relationships between the variables and consequently determine the effect of mediators on such relationships with more accuracy. Additionally, the inclusion of both components of SWB is also an important element to consider in future studies. As for self-transcendent aspirations, studies considering the self-transcendent vs. physical goals axis are still scarce.

One of the strengths of our article is its large sample size, which allows for detecting effect sizes as small as *f*^2^ = 0.006, which helps minimize type II errors. However, a note of warning should be made. Because of our large sample size and its high power, it is very likely that almost all standardized paths in our models become significant irrespective of its strength. Indeed, our paths range from 0.02 (small) to 0.77 (strong), according to Richard et al. (2003). Therefore, “significance” is important, but readers are also encouraged to pay attention to the strength of each path.

## Conclusion

First, our results show that self-transcendent life aspirations are strongly related to life satisfaction. This is relevant because of the following reasons: (i) currently, there is great interest in the relevance of prospective thinking for human beings, understanding that it is a motivational driver crucial to guide cognitive and affective processes (Forgas and Baumeister, 2018); (ii) over the last years, there has been growing interest in classifying intrinsic aspirations considering the categories physical vs. self-transcendent proposed by Grouzet et al. (2005). However, few studies have explored the relationship between self-transcendent aspirations, such as community contribution, and life satisfaction.

Although aspirations such as long-term goals can be relevant for the human being, the results show the interplay between different mechanisms for elucidating how aspirations focused on self-transcendence can increase life satisfaction. In this sense, self-transcendent aspirations promote the experience of gratitude, which, in turn, has a total mediating effect over life satisfaction. Thus, gratitude, as a trait emotion, plays a key role in the satisfaction that people experience with social relationships and contributes to the experience of positive affect in daily life, as well as nurturing personal resources (Fredrickson, 2004).

Another fundamental aspect is the role of empathy in these relationships and, specifically, in people with high levels of self-transcendent aspirations and high cognitive empathy. However, no moderating role is observed in the case of affective empathy. This confirms the relevance of a more rational perspective-taking for an increase of SWB.

From the perspective of developmental psychology, our results emphasize the importance of life aspirations focused on community well-being at schools. Understanding and internalizing the relevance of these long-term self-transcendent goals can encourage prosocial behavior in children and adolescents by means of emotions such as gratitude, as well as contribute to their life satisfaction.

## Data Availability Statement

The raw data supporting the conclusions of this article will be made available by the authors, without undue reservation, to any qualified researcher.

## Ethics Statement

The studies involving human participants were reviewed and approved by the Comité de Ética en la Investigación de las Facultades de Humanidades y de Derecho de la Universidad Continental. The patients/participants provided their written informed consent to participate in this study.

## Author Contributions

XO developed the original idea and design of the research. RM contributed to the collection of the information. XO and JU wrote the article concerned with the writing and interpretation of results. JU, RM, and AA analyzed the data. AA and CB were in charge of the review. All authors read, reviewed, and approved the final manuscript.

## Conflict of Interest

The authors declare that the research was conducted in the absence of any commercial or financial relationships that could be construed as a potential conflict of interest.
